# Genome assessment of *Lactobacillus acidophilus* WG8, a putative probiotic strain isolated from Holstein-Friesian raw milk

**DOI:** 10.1128/mra.01255-25

**Published:** 2026-03-12

**Authors:** Sinalo Mani, Tshifhiwa Paris Mamphogoro

**Affiliations:** 1 Gastrointestinal Microbiology and Biotechnology Unit, Animal Production Institute, Agricultural Research Council, Irene, Pretoria, South Africa; Nanchang University, Nanchang, Jiangxi, China

**Keywords:** genome assembly, *Lactobacillus acidophilus*, dairy microbiota, probiotic, gut health

## Abstract

This study reports the draft genome sequence of *Lactobacillus acidophilus* strain WGS8 isolated from cow raw milk. This strain exhibits genetic features associated with probiotic functionality. The draft genome is 1,957,515 bp in length, assembled into 16 contigs.

## ANNOUNCEMENT

*Lactobacillus acidophilus* is a gram-positive, homofermentative bacterium commonly found in the gastrointestinal tracts of humans and animals, as well as in fermented dairy products (1, 2). As a core member of the gut microbiota, it contributes to intestinal homeostasis through the production of lactic acid, bacteriocins, and other antimicrobial compounds that inhibit enteric pathogens (3, 4). The probiotic effects of *L. acidophilus* are strain-specific, including enhanced gut barrier integrity, modulation of immune responses, and beneficial impacts on microbial community composition (5, 6). Its physiological resilience, such as acid and bile tolerance and survival in dairy matrices, supports its broad application in functional foods and probiotic formulations (7, 8).

Raw milk samples were collected from Holstein-Friesian dairy cows maintained at the Agricultural Research Council (ARC) Dairy Section, Irene, South Africa (GPS: –25.892, 28.222). Ten-fold serial dilutions of each sample were prepared using sterile phosphate-buffered saline (PBS, pH 7.2), and aliquots were spread-plated onto de Man, Rogosa, and Sharpe (MRS) agar (Oxoid, UK) supplemented with 0.05 g/L cysteine-HCl. Plates were incubated anaerobically using an Anaerobic Gas Pack System (Oxoid, UK) at 37°C for 24 to 48 h. Distinct colonies with morphological characteristics typical of *Lactobacillus* spp. were selected and purified by repeated streaking on MRS agar until a single colony morphology was obtained. Single colonies were obtained by subculturing onto MRS agar plates, and then inoculated into MRS broth at 37°C for 24 h for DNA extraction.

Genomic DNA was extracted from pure cultures using a Bacterial Miniprep Kit (Zymo Research, Irvine, CA, USA). DNA concentration and quality were evaluated using NanoPhotometer NP80 (Implen GmbH, Munich, Germany) and a Qubit fluorometer (Thermo Fisher Scientific, MA, USA). Genomic DNA was used to prepare library preparation with the MGIEasy Universal DNA Library Prep Set (MGI Tech, Shenzhen, China). Whole-genome sequencing was conducted on the DNBSEQ-G400 MGI platform (MGI Tech, Shenzhen, China) using paired-end sequencing (2 × 150 bp) chemistry. Quality control of raw reads was assessed using FastQC v0.72 (http://www.bioinformatics.babraham.ac.uk/projects/fastqc), and low-quality reads were trimmed using Trimmomatic v0.36 (9), retaining bases with phred quality scores of 30 or higher for 90% of the read length. This yielded 4,831,143 high-quality reads, achieving ~610× genome coverage. The reads were assembled *de novo* using SPAdes v3.15.3 (10), and assembly quality was evaluated using QUAST v5.0.2. Genome completeness was assessed using CheckM v1.0.18 (11) and estimated to be 98.94% at the genus level.

The resulting draft genome comprised 1,957,515 bp across 17 contigs (N50 = 231,424 bp), with a guanine-cytosine (GC) content of 34.5%. Genome annotation was carried out using PGAP (12) and identified 1,905 total genes, of which 1,783 were protein coding. Taxonomic assignment using GTDB-Tk v2.3.2 (13) confirmed the isolate as *Lactobacillus acidophilus*. The genome shared an average nucleotide identity (ANI) of 99.97% with *Lactobacillus acidophilus* strain DSM 20079 (GCA_003047065.1), as calculated by FastANI v0.1.3 (14), confirming its affiliation with the *Lactobacillus* taxon. Compared with DSM 20079 (2.01 Mb, one CRISPR locus), strain WGS8 has two CRISPR arrays and fewer pseudogenes (61 vs 99). These differences suggest the presence of accessory genomic elements and strain-specific adaptive features, supporting that WGS8 represents a distinct strain within the *L. acidophilus* species complex. All software programs were run with default parameters. Gene annotation revealed the presence of genes involved in adhesion, acid tolerance, bile salt tolerance, and immunomodulation, all associated with probiotic activities. Additionally, the genome was found to contain 58 tRNAs and 3 ncRNAs.

## Data Availability

This whole-genome sequencing project has been deposited at NCBI under BioProject number PRJNA1262373. The version described in this paper is the first version, with a genome accession number of GCA_050573395.1. The SRA accession number is SRR33598564, and the BioSample accession number is SAMN48580605.
